# Editorial: Bioengineering for spinal cord injury

**DOI:** 10.3389/fnhum.2026.1943147

**Published:** 2026-07-31

**Authors:** Munehisa Shinozaki, Syoichi Tashiro

**Affiliations:** 1 Keio University Regenerative Medicine Research Center, Kanagawa, Japan; 2 Department of Rehabilitation Medicine, Kyorin University School of Medicine, Tokyo, Japan

**Keywords:** bioengineering, electric stimulation, neural plasticity, spinal cord injury, tissue clearing

Every year, 930,000 people suffer from spinal cord injury (SCI), often resulting in irreversible sequelae (GBD 2016 Traumatic Brain Injury Spinal Cord Injury Collaborators, 2019). Because the spinal cord, like the brain, has extremely limited regenerative capacity (Ahuja et al., 2017), rehabilitation remains a main therapeutic approach for improving functional impairments of SCI patients. Currently, new treatments are being researched worldwide, and many clinical trials are underway. In parallel, recent advances in bioengineering are also shedding new light on this intractable disease through various mechanisms (Courtine and Sofroniew, 2019).

One mechanism is the activation of residual neural circuits. Since neural activity depends on electrically excitable cells and synaptic signaling, neural circuits are able to be modulated by applied electric fields or by currents induced through magnetic stimulation. Over the past decade, electrical stimulation of the spinal cord has attracted substantial attention in neuromodulation for SCI (Wagner et al., 2018). It is now also understood that the brain undergoes dramatic functional and structural changes following SCI (Matsubayashi et al., 2023), and it has been reported that spinal cord function may be promoted by interventions that engage supraspinal plasticity. Furthermore, skeletal muscles below the injury are controlled by spinal motor neurons, while proprioceptive signals from muscle spindles and other sensory receptors provide afferent feedback to spinal circuits (Zieriacks et al., 2021). Peripheral nerve or muscle stimulation can therefore modulate the excitability of residual spinal networks.

Another bioengineering approach involves intervening at the injury site with the goal of regenerating damaged tissue. For example, after SCI, a cavity is formed at the injury site, which inhibits regeneration in humans; therefore, transplanting biomaterial scaffolds with high tissue affinity into this cavity may induce axonal outgrowth in the host's neural tissue (Koffler et al., 2019). In another example, while the central nervous system is normally isolated from the vascular system by the blood-brain barrier and the blood-spinal cord barrier, engineering techniques have been developed to deliver specific drugs across these barriers (Terstappen et al., 2021).

In addition, new platform technologies for basic research aimed at developing the treatments described above are, in a broad sense, also included in the category of bioengineering for SCI. Specifically, these involve the creation of animal models of spinal cord injury, the *in vitro* and *in silico* replication or simulation of the spinal cord and its pathological states, and systems for visualizing the central nervous system, as well as for evaluating their phenotypes. In recent years, techniques for mimicking the spinal cord using organoids, spheroids, and assembloids have been reported (Kim et al., 2025).

For this topic, we broadly solicited submissions on bioengineering for SCI, and four papers were published. First, Buxton et al. reported on sciDISCO, a tissue clearing method for three-dimensional evaluation of mouse spinal cord in basic research. While numerous tissue-clearing techniques have been reported in the past, Buxton et al. extended existing clearing workflows by using a 3D printer to create a straightener to prevent the spinal cord from bending during the clearing process. Furthermore, because the meninges can restrict tissue expansion and impair clearing and antibody penetration in most protocols, they are often removed before spinal cord clearing. In contrast, sciDISCO enabled clearing and immunolabeling with the meninges intact, allowing visualization of meningeal lymphatic vessels. This report presents a practical protocol for the DISCO system in mouse injured spinal cord.

The other three papers in this topic all involve electrical stimulation of the human spinal cord. Shankar and Chandran reported a protocol for a randomized controlled trial targeting chronic incomplete spinal cord injury. The protocol is designed to test whether intermittent theta burst stimulation (iTBS)-induced modulation of cortical excitability, combined with transcutaneous spinal cord stimulation (tSCS)-mediated modulation of spinal interneuronal networks and motor pools, enhances corticospinal plasticity and lower-limb motor output more effectively than tSCS alone. Chu et al. reported on a small-scale randomized controlled trial involving 25 patients with incomplete spinal cord injury 1–6 months post-injury, in which the brain, the area near the spinal cord surrounding the level of injury, and the sciatic nerve were stimulated simultaneously, and the outcome measures were evaluated after 8 weeks. A distinctive feature of this study is that, in addition to the stimulation of three anatomically distinct targets, traditional acupuncture needles were used as deep-stimulation electrodes, and an ultrasound device was used to identify the targets and determine the required needle-insertion depth. Acupuncture needle electrical stimulation can be described as a minimally invasive nerve stimulation method that may be viewed as an intermediate approach between surface electrodes and implantable electrodes. Finally, Upadhyay et al. conducted a systematic review examining the effects of spinal cord stimulation and nerve root stimulation on autonomic dysfunction following SCI. After SCI, orthostatic hypotension, autonomic dysreflexia, urinary dysfunction, bowel dysfunction, and sexual dysfunction in addition to sensorimotor paralysis significantly affect not only patients' ADLs but also their QOL. This review includes 151 cases of epidural spinal cord stimulation (eSCS), 48 cases of tSCS, and 1,249 cases of sacral anterior root stimulation (SARS) from 57 reports. In response to acute stimulation, eSCS/tSCS tended to increase blood pressure and alleviate orthostatic hypotension, and showed a tendency to improve bladder capacity and voiding efficiency, as well as to shorten bowel management time. eSCS or SARS tended to induce or improve erections.

This topic featured four contributions that offered a variety of insights. Although more than 10 years have passed since the breakthrough in tissue clearing, the fact that there is still room for improvement in tissue-clearing protocols in spinal cord injury research suggests that three-dimensional evaluation will become even more widespread in the future, leading to deeper insights. Meanwhile, the fact that reports on spinal cord stimulation accounted for a large proportion of this topic mirrors the growing clinical interest in neuromodulation. In this field, the compatibility of percutaneous stimulation compared to epidural stimulation using surgically implanted electrodes has drawn attention, thereby offering patients a wider range of options. Furthermore, as discussed above, synergistic effects with supraspinal and peripheral stimulation are also anticipated, and it is realistic to expect that this will become a standard treatment performed in combination with rehabilitation to activate residual tissue in the near future, at least for selected patients.
